# Acute effect of a multi-ingredient pre-workout supplement on pacing and kinetic expression during shorter and longer bouts of high intensity functional training

**DOI:** 10.1080/15502783.2025.2529906

**Published:** 2025-07-08

**Authors:** Gerald T. Mangine, Christopher Staples, James W. Henley, Ashley Hines, Kristyn C. McGeehan, Wysmark Chaves, Wil King, Tiffany A. Esmat, John R. McLester, Jacob L. Grazer

**Affiliations:** Kennesaw State University, Exercise Science and Sport Management, Kennesaw, Georgia

**Keywords:** CrossFit®, HIFT, AMRAP, repetition completion rate, force, power

## Abstract

**Background:**

High-intensity functional training (HIFT) varies daily workout programming, and trainees are often tasked with completing “as many repetitions as possible” (AMRAP) within a set time. Successful performance depends on fatigue management skills and maintaining a consistent expression of force and velocity when performing and transitioning between exercises over the duration of the workout. Multi-ingredient pre-workout (MIPS) supplement formulations often contain ingredients shown to facilitate energy availability and mitigate fatigue, and therefore, might positively affect HIFT performance. This study aimed to assess a multi-ingredient pre-workout supplement’s acute effect on pacing and the expression of kinetics throughout a 5- and 15-minute HIFT-style AMRAP.

**Methods:**

Twelve men and 10 women (29.3 ± 7.1 years, 171 ± 7 cm, 80.5 ± 15.6 kg) with HIFT experience ( > 2 years) completed four weekly visits, consuming either the supplement (S) or placebo (P) before a 5- or 15-minute AMRAP of rowing (9 or 7 Calories for men and women), six barbell thrusters (43.1 kg and 29.5 kg), and three jumps onto a box (0.61 m and 0.51 m). Video recordings of each workout were analyzed to quantify repetitions completed, volume load (kg), and pace (i.e. time spent on each workout component, repetition completion rate). The expression of kinetics and related factors were quantified during rowing via the ergometer microcomputer (Calories per stroke, power [W], and strokes per minute), during barbell thrusters by a 3D motion tracking system (barbell velocity [m · sec^−1^] and power [W]), and by in-ground force plates during box jumps (peak and mean force [N], time-to-peak force [ms], rate of force development [RFD; N·sec^−1^], and impulse [N·sec]). The averages, standard deviation, and slope across rounds were calculated for all pacing and kinetic variables for separate 3-way (sex × workout durations × supplement conditions) repeated measures analysis of variance comparisons.

**Results:**

Men and women completed a greater total volume load (~4.4%, *p* = 0.009) and repetitions at a faster rate (~2.2%, *p* = 0.043) during S compared to P and regardless of workout duration. These were best explained by participants completing a greater rowing volume load (~8.1%, *p* = 0.009) by averaging more powerful strokes (~7.0%, *p* = 0.005), more consistent transitions to rowing (~11.0%, *p* = 0.020), and a trend for faster barbell thruster repetition completion rate (~2.3%, *p* = 0.077). Interestingly, participants varied more across rounds during S (~16.8–29.4%, *p* < 0.05) in box jump force expression (peak force, mean force, time-to-peak force, and RFD). No other differences were seen between supplement conditions, only expected differences were seen between men and women and between workout durations.

**Conclusions:**

Although it did not affect repetitions completed, the multi-ingredient pre-workout supplement led to a greater volume load and faster overall repetition completion rate. These were primarily due to improvements surrounding rowing performance and evidence of an effect on thruster pacing and box jump force expression. The effects were similar in both sexes and workout durations. Athletes might consider this MIPS formulation to improve HIFT performance, particularly when a workout contains a component that requires sustained, continuous effort.

## 1. Introduction

High-intensity functional training (HIFT) incorporates multiple exercise modalities into a single “workout of the day” (WOD) for the purpose of eliciting comprehensive adaptations in fitness [1,2]. Although the exact prescription (i.e. absolute and relative intensity, volume, density, etc.) for each exercise within a WOD can vary, trainees are typically encouraged to put forth a high-intensity or vigorous effort (heart rate ≥ 85% max, lactate ≥13.3 mmol/L) [1,2]. For instance, the most common WOD design organizes exercises into a circuit and challenges trainees to repeat the circuit while completing as many repetitions as possible (AMRAP) within a set time limit [1,3,4]. Regardless of whether the format follows an AMRAP or some other design, the underlying instruction is usually to complete more work in less time by maintaining the fastest pace possible.

Consistently completing repetitions at a faster rate while minimizing time breaking and transitioning between exercises should enable more repetitions to be completed within the WOD’s duration. However, the actual pace employed on each movement and the overall workout are dictated by the trainee’s experience and familiarity with assigned exercises, the relative (or perceived) difficulty of those exercises at assigned volume-loads, the assigned or expected duration of the WOD, individual fitness in relevant physiological parameters, and accumulated fatigue [4–6]. Some of these may become less influential in more experienced trainees, who would have been exposed to and acquired more skill in a greater variety of movement patterns than a novice [7,8]. Their consistent training would also have led to physiological adaptations in relevant attributes [9], and potentially helped them learn strategies to limit the accumulation of fatigue during a WOD [10,11]. Thus, it may be assumed that WOD performance by a trained individual would predominantly be limited by their physiological attributes and fitness.

Video recorded efforts are commonly required for verification purposes in HIFT competition [12], but they can also be used to evaluate technique and the success of a pacing strategy [4–6,13]. Previous studies have done this by averaging pacing elements (i.e. repetition, break, and failed repetition counts, time spent on sets, breaks, and transitions) over the entire WOD and by calculating their slope and standard deviation (SD) across sets/rounds or minutes [5,6,13]. Averages (e.g. average repetition completion rate) provide an overall estimate of each element and are primarily affected by the relative intensity and duration of the WOD, individual fitness in relevant physiological attributes, and skill in devising a matching pacing strategy [4]. Strategy execution can be further elucidated by the slope and SD of each element. For instance, a negative slope in repetition completion rate would reveal an aggressive strategy that allowed fatigue to limit performance toward the end of the WOD. Meanwhile, a slope of zero could imply a more consistent pace but consistency is more precisely identified by a lower SD [4]. Assuming sufficient experience and familiarity with programmed exercises, individual fitness would be most responsible for limiting fatigue and enabling a faster, more consistent effort. However, this can only be assumed with skepticism when deriving pacing elements from a video. Estimating work and its rate of completion (i.e. power) is extremely imprecise when compared to more direct measurement [4]. Pairing video analysis with technology that is capable of monitoring kinetics provides a more accurate assessment of the influence of fatigue on WOD performance.

Although consistent exercise provides the stimulus for improving factors related to endurance (e.g. availability and utilization of energy substrates, muscle buffering capacity) [14–16], these traits may be acutely enhanced through nutritional supplementation [11,17]. Multi-ingredient pre-workout supplements (MIPS) are a popular class of dietary supplements that are often formulated with ingredients that aim to promote blood flow and energy availability, the expression of force and power, and the attenuation of fatigue during exercise [18]. Although ingredient profiles and doses differ between products, the most common MIPS ingredients include: caffeine, creatine, beta alanine, branched chain amino acids, and those known to stimulate endogenous nitric oxide production (e.g. L-citrulline, beet root extract, red spinach extract) [19]. A great deal of attention has been placed on the ergogenic benefits provided by several individual ingredients or in limited combinations, but not when consumed as part of a MIPS formulation. Most of these studies have looked at the effect of MIPS on single-modality performance. Some have noted improved volume-load completed in resistance training exercise [20,21], aerobic and anaerobic capacity [22], and time-to-exhaustion in cardiorespiratory exercise [21,23,24] at reduced perception of fatigue [21], whereas others did not see improvements in vertical jump performance [25] or measures of upper body strength and power [26]. From these, MIPS appear more likely to be beneficial when the physical task challenges or requires sustained energy availability.

To the best of our knowledge, only one study has examined the effects of any MIPS formulation on HIFT performance [22]. Outlaw and colleagues (2014) had participants consume a blend of pomegranate, tart cherry, beet root, and green tea extracts 30 minutes prior to training sessions (3–5 sessions per week) for six weeks. Performance adaptations were evaluated by an initial, time-to-completion WOD that typically lasted 7.9 minutes, followed by a 20-minute rest, and then a 15-minute AMRAP that favored the MIPS group at the end of the study. Although this demonstrates a benefit, it is not clear why there was no effect on the first WOD. The duration and energy required of the first WOD may not have been sufficient compared to the combination of two WODs, though a few sources of variability may have masked the true effect. There could have been more variability in the volume load completed by men compared to women. Both test WODs incorporated exercises that typically scale intensity-loads between sexes [3], but the authors did not report whether this happened, nor did they make any apparent attempt to equate volume loads. Additionally, the participants’ HIFT experience (i.e. at least 6 months of regular training) was on the low-end of what has been defined as HIFT-trained ( > 1–2 years) [1]. Less experience may have led to a variety of strategic approaches to both test and training WODs, and this could have been compounded by the study’s requirement of maintaining normal training habits. Those who regularly trained more often would have been exposed to more workouts, had more opportunities to refine pacing strategies, and would have consumed the MIPS more often due to study instructions. Furthermore, it remains unknown whether MIPS acutely impacted WOD performance.

The purpose of this study was to examine the acute effects of MIPS and exercise duration on exercise kinetics expression during an AMRAP-style HIFT workout in experienced men and women. Since a single serving of the present formulation improved repetition-volume completed in resistance training exercise [20], it was hypothesized that a greater volume load would be completed, and the expression of kinetics would be more consistent, following consumption of MIPS in both shorter- and longer-duration HIFT efforts. Kinetic expression was expected to be less during the longer-duration bouts to sustain effort over more time [16], and thus, differences between supplement conditions were hypothesized to be greater with the longer duration bouts. Finally, effective dosages for individual nutritional supplement ingredients are often made relative to body mass. However, because dosages within commercially available MIPS formulations are based off a standard body mass, it was hypothesized that the supplement would be more beneficial in women due to the likelihood of them having a lower body mass [27,28] and receiving a higher relative dosage.

## 2. Materials and methods

### 2.1. Participants

Based on a previously observed effect of *f* (0.33) [20] and the following parameters (*p* < 0.05; β = 0.80) for four conditions completed in cross-over fashion, a priori analysis indicated that a minimum of 14 participants were needed for this investigation. Following a thorough explanation of the study design, procedures, and supplement ingredients, 23 men and women, with at least 2 years of HIFT experience provided their written informed consent to participate in this double-blind, placebo-controlled study. All participants were free from any injury or illness that could have impacted their performance and were not using any performance enhancing drugs or medications (as determined by PAR-Q+ and Health and Medical History Questionnaire). During the study, one male participant was removed due to scheduling issues that prevented him from completing his final visit within study timeline requirements. All other participants completed all study visits and reported no adverse events. The physical and performance characteristics measured at baseline and training experience metrics of the final sample are presented in Table 1. Men were significantly (*p* < 0.05) taller, weighed more, possessed more lean mass and less body fat mass, and outperformed women in each performance assessment except vertical jump height. Age and training experience were similar between men and women, except women possessed approximately 3.4 more years of regular (i.e. > 2 training sessions per week) HIFT training experience (*p* = 0.015). Overall HIFT experience was the same between men and women. This study was approved by the University Institutional Review Board (#IRB-FY23-18).

**Table 1.** Participant demographics.

	Men	Women	Total
Age (years)	27.6 ± 8.0	31.3 ± 5.5	29.3 ± 7.1
Height (cm)	175 ± 7	167 ± 5‡	171 ± 7
Body mass (kg)	89.1 ± 12.1	70.2 ± 13.2‡	80.5 ± 15.6
Body fat (%)	15.8 ± 5.9	20.9 ± 6.2	18.1 ± 6.5
Lean mass (kg)	74.8 ± 9.3	55.3 ± 9.5‡	65.9 ± 13.5
Estimated Thruster 1-RM (kg)	90.3 ± 16.3	57.2 ± 11.8‡	74.8 ± 21.8
Estimated Relative Thruster strength (per kg)	1.02 ± 0.16	0.81 ± 0.09‡	0.92 ± 0.17
2K Rowing Time (minutes)	7.47 ± 0.41	8.52 ± 0.54‡	7.95 ± 0.71
Vertical Jump Height (m)	0.53 ± 0.18	0.45 ± 0.11	0.49 ± 0.15
Resistance Training Experience (years)	12.3 ± 7.4	9.6 ± 2.3	11.1 ± 5.8
Regular Resistance Training (years)	10.7 ± 7.8	8.6 ± 1.8	9.8 ± 6.0
Gymnastics Experience (years)	1.3 ± 1.9	2.1 ± 3.2	1.6 ± 2.5
Gymnastics Competition Experience (years)	0.5 ± 1.3	0.6 ± 1.3	0.6 ± 1.3
HIFT Experience (years)	4.9 ± 2.2	7.1 ± 3.2	5.9 ± 2.8
Regular HIFT Training (years)	3.5 ± 1.5	6.9 ± 3.2‡	5.1 ± 2.9

Regular was defined as > 2 training sessions per week for most of the year (≥8 months). ‡ = Significant (*p* < 0.05) difference between men and women.

### 2.2. Study design

Participants were asked to report to the Human Performance Laboratory on five separate occasions within a 5–6-week period. The first baseline visit had participants complete body composition assessments followed by vertical jump height, barbell thruster strength, and 2,000-m rowing for time testing. The remaining four visits were randomized experimental sessions that had participants consume a formulation containing either the supplement (S) or placebo (P), rest 40-minutes, and then begin a standardized warm-up protocol. The participants were then informed of whether they were assigned to complete either a 5- or 15-minute AMRAP workout. The cross-over design ensured that each participant completed all possible supplement-workout combinations (i.e. 5-minute AMRAP with S or P [5S or 5P], 15-minute AMRAP with S or P [15S or 15P]) over the course of the study. All visits occurred on the same day of the week at a time of day that was consistent with each participant’s normal workout time. Participants were asked to arrive for each visit 2–3 hours post prandial, maintain their normal dietary habits for the duration of the study (verified by 3-day food logs), and to refrain from vigorous exercise for at least 48 hours prior to any visit. Research questions were answered by making comparisons between men and women, workout durations, and supplement condition.

### 2.3. Baseline testing

Participants began testing with an initial measurement of height (±0.1 cm) and body mass (±0.1 kg) while wearing athletic attire and standing barefoot on a stadiometer (WB-3000, TANITA Corporation, Tokyo, Japan). Subsequently, body composition was assessed by three methods (i.e. dual-energy X-ray absorptiometry [iDXA, Lunar Corporation, Madison, WI], air displacement plethysmography [BodPod, COSMED USA Inc., Chicago, IL], and bioelectrical impedance analysis [770 Body Composition and Body Water Analyzer, InBody, Seoul, South Korea]) using previously described, standard procedures [29]. Body mass, bone mineral content (BMC; from iDXA), body volume (from BodPod), and total body water (from bioelectrical impedance analysis) were entered into a 4-compartment model (Equation 1), to estimate body fat percentage (BF%) [30] and fat-free mass (±0.1 kg).

*Equation 1*:

$$\mathrm{BF}\%=\frac{\left(2.748\;x\;\mathrm{volume}\right)-\left(0.699\;x\;\mathrm{water}\right)+\left(1.129\;x\;\mathrm{BMC}\right)-\left(2.051\;x\;\mathrm{BodyMass}\right)}{\mathrm{Body}\;\mathrm{Mass}}x100$$

After body composition assessment, participants were allowed to consume a light snack (e.g. granola bar) before completing performance testing. These began with a standardized warm-up consisting of 5 minutes on a rowing ergometer followed by a series of dynamic stretches (10 repetitions of arm circles, alternating body hugs, walking knee hugs, walking quad pulls, and walking lunges) and workout-specific exercises (10 repetitions of body squats, 10 barbell thrusters with an unloaded barbell [women = 15.9 kg; men = 20.4 kg], 10 unweighted box step-ups, and 10 box jumps with step-down [box height = 0.51 m for women; 0.61 m for men]). Participants then completed testing in the following order to limit the impact of fatigue on subsequent tests: vertical jump height, barbell thruster strength, and 2,000-m rowing for time. All performance tests were completed in the presence of a certified strength and conditioning specialist (CSCS). Verbal encouragement was given on all maximal tests.

Vertical jump height testing was performed using a testing station (Vertec, JUMPUSA, Sunnyvale, CA, USA) that consisted of a weighted base and a distance-marked, extendable vertical neck. The uppermost 24-inch portion of the vertical neck was aligned with horizontal tabs at 1-inch intervals that rotate when tapped. The test began by having participants reach overhead with their dominant hand to tap the highest possible horizontal tab while keeping their heels on the floor. The neck was then extended, and participants were then allotted 3–5 maximal counter movement jumping attempts, separated by at least 1 min of rest, to again reach the highest horizontal tab possible. Vertical jump height was calculated as the difference between maximal jump height and maximal vertical reach in inches and then converted to meters (m).

Barbell thruster strength was estimated from a 3–5 repetition-maximum (RM) thruster assessment. Participants began by completing a submaximal warm-up set at approximately 60–70% of their estimated 1-RM for 4–6 repetitions, rested 2–4 minutes, and then completed another warm-up set of 1–3 repetitions at approximately 80–90% of their estimated 1-RM. Following a 3–5-minute rest, they were allotted two maximal trials to find the highest load they could complete 3–5 repetitions of the barbell thruster exercise while maintaining the technical standards employed in official HIFT competition [31]. Technical standards were verified by a researcher positioned to the side of the participant. All barbell thruster sets began from the floor and 5 min of rest was allotted if a second trial was necessary. After determining the 3–5-RM, 1-RM was estimated via the Brzycki estimation formula [32].

The 2,000-m rowing test was completed on a rowing ergometer (model D, Concept2 Inc, Morrisville, VE) using its microcomputer to set a 2,000-m countdown to initiate starting with the participant’s first pull. The test concluded and time (in minutes) was automatically recorded when the counter reached 0 m. For this assessment only, participants were permitted to self-select their 0–10 damper setting.

### 2.4. Supplementation protocol

Participants ingested either S or P mixed with 10 fl. oz. of water upon arrival on each experimental visit. The S formulation was a commercially available, MIPS supplement (SHIFTED Maximum Formula Pre-Workout, SHIFTED LLC, Monteagle, TN, USA) containing a blend of ingredients shown to assist in energy availability (see Table 2). The P formulation consisted of one serving of commercially available, non-caloric water flavoring drops (MiO Fit Liquid Water Enhancer – Berry Blast; Kraft Heinz Co., Chicago, IL, USA), and was similar to S in taste and appearance. Participants imbibed S and P from a nontransparent shaker bottle while wearing a nose clip to reduce their ability to notice any differences in taste, smell, or appearance. The efficacy of these precautions was evaluated by asking participants to guess whether they consumed S or P immediately following ingestion and again after exercise and then explain their choice. The researchers requesting this information did not know which answer was correct at either time point. To maintain a double-blind design, a single researcher was made responsible for randomly assigning and preparing S and P prior to each study visit, and then placing mixed shaker bottles in a laboratory refrigerator (at approximately 1.7° C) until participants arrived. To avoid any influence this researcher might have had on participant performance or the other researchers, this researcher had no other responsibilities during experimental sessions. Nevertheless, participants correctly identified S and P approximately 78% of the time both before and after each workout, but they were not informed of this until after they had completed all study visits. The difference in “texture” and a “tingling feeling” were the most common reasons given prior to and post-exercise, respectively. To be consistent with previous studies [20,25], participants rested 40 minutes after consuming S or P before initiating the standardized warm-up.

**Table 2.** Supplement ingredients.

Serving Size: 1 scoop (30 g)		
**Ingredients**	**Amount per serving**	**% DV**
Calories	5	
Total Carbohydrate	1 g	≤1%*
Niacin (as Nicotinic Acid)	15 mg	94%
Vitamin B6 (as Pyridoxine HCl)	1 mg	59%
Vitamin B12 (as Methylcobalamin)	100 mcg	4167%
Iron	1 mg	6%
Magnesium (from Red Spinach Leaf Extract and Dimagnesium Malate)	9 mg	2%
Sodium (as Pink Himalayan Sea Salt)	40 mg	2%
Potassium (from Red Spinach Leaf Extract and Potassium Chloride)	248 mg	5%
L-Citrulline	8 g	**
Creatine Monohydrate	5 g	**
Taurine	3 g	**
Beta-Alanine (as CarnoSyn®)	2.5 g	**
Betaine Anhydrous	2.5 g	**
L-Tyrosine	2 g	**
Red Spinach Leaf Extract (as Oxystorm®)	1 g	**
Beet Root Extract	1 g	**
Alpha-GPC (Alpha-Glycerol Phosphoryl Choline 50%)	300 mg	**
Caffeine Blend Caffeine Anhydrous (250 mg) zümXR® Delayed Release Caffeine (50 mg)	300 mg	**
L-Theanine	150 mg	**
ElevATP® (Ancient Peat and Apple Fruit Extract)	150 mg	**
Pink Himalayan Sea Salt	100 mg	**
Rhodiola rosea (root) Extract	100 mg	**
Co-Enzyme Q10	25 mg	**
AstraGin® [Astragalus membranaceus (root) Extract & Panax notoginseng (root) Extract]	25 mg	**
BioPerine® (Black Pepper Fruit Extract)	5 mg	**

*Percent Daily Values (DV) are based on a 2,000-calorie diet.

** Daily value not established.

Other ingredients: Citric acid, Natural Flavor, Calcium Silicate, Malic Acid, Silicon Dioxide, Sucralose, Spirulina Powder.

### 2.5. Workout protocol and kinetics assessment

**Figure 1.**
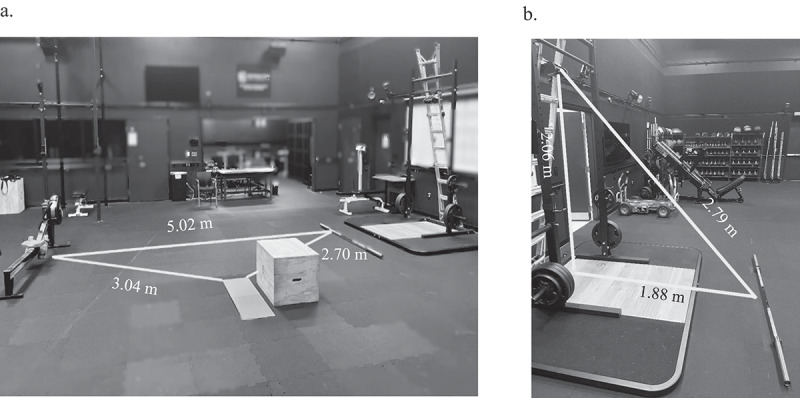
Standardized position of (a) exercise station and (b) 3D motion camera to barbell.

All experimental sessions were supervised by a CSCS. Participants followed the same standardized warmup as used during baseline performance testing, rested for 5 min, and then completed either 5- or 15-minute workout protocol. Regardless of duration, the workout consisted of the same AMRAP circuit of rowing on an ergometer (9 Calories per round for men, 7 Calories per round for women), six barbell thrusters (43.1 kg for men, and 29.5 kg for women), and three box jumps to a standard height (0.61 m for men and 0.51 m for women) with mandatory step down. These exercises and their differential prescription for men and women were selected because they commonly appear in HIFT [3,31,33] and enabled the collection of movement kinetics using our laboratory’s available technology. Stations for each exercise were placed in sequential fashion at standard distances to avoid any influence variations might have on repetitions completed within fixed workout durations (see Figure 1(a)). All experimental trials were scored as total rounds and repetitions completed, round- and repetition-completion rate (per minute), total volume-load completed (i.e. sum of rowing, thruster, and box jump volume-loads), and total failed repetitions. To ensure accuracy and quantify pacing elements, all experimental trials were video recorded by a 3.5-megapixel Microsoft Surface 3 tablet camera (Microsoft Corp., Redmond WA) at 1,920 х 1,080 pixels/30 frames per second using previously described positioning standards [34]. Video recordings were analyzed to verify performance scores and quantify the average, slope, and SD across all rounds for repetitions completed and failed for each exercise, breaks taken, and time (in seconds) spent on breaks and transitions [5,6,13].

Rowing was completed on the same ergometer as used during baseline testing. All trials were completed using a standard damper setting at 10 to avoid any influence preferential setting might have on Calories rowed per stroke between and within participants. To ensure the exact number of Calories were rowed on each round, the rowing ergometer microcomputer was set to the interval-Calorie function with prescribed calories (i.e. 9 for men and 7 for women) and rest time set at indefinite (i.e. 10-minute maximum). A researcher located near the ergometer performed the required manual restart for each round when participants were completing box jumps at the end of each preceding round. At the completion of the workout, the data was saved on the ergometer and transferred via flash drive to the Concept2 logbook application. To quantify performance during the rowing aspect of the AMRAP, total rowing Calories (i.e. repetitions), rounds, duration (in minutes), distance (in meters), and strokes were retained from the application. Total distance, strokes, duration, average watts, and average Calories per stroke were also collected on each round, and then used to estimate volume-load (in kg) by converting watts to kgm *x* min^−1^ (1 watts = 6.118 kgm *x* min^−1^), factoring out distance and duration, and then multiplying by strokes per round (see Equation 2). The average, slope, and SD of each of these across the workout duration were retained for comparisons.*Equation 2*:

$$\mathrm{Rowing}\;\mathrm{volume}\;\mathrm{load}\left(\mathrm{kg}\right)=\frac{\left(6.118\;x\;\mathrm{Average}\;\mathrm{Watts}\right)\;x\;\mathrm{Total}\;\mathrm{Strokes}\;x\;\mathrm{Duration}\;\left(\mathrm{minutes}\right)}{\mathrm{Distance}\;\left(\mathrm{meters}\right)}$$

The collection of barbell thruster kinetics has been described in detail elsewhere [34]. Kinetics were monitored by a three-dimensional motion-tracking camera (PERCH, Catalyft Labs, Inc., Cambridge, MA, USA) that was Velcro-strapped to a ladder placed behind a power rack. To ensure all repetitions were completed at the same approximate distance from the camera, a line of tape was placed on the floor parallel to the ladder for the duration of the study (see Figure 1(b)). Research assistants initially set, and then reset between rounds, the shaft of the loaded barbell directly above this line. Participants could then walk up to the line and immediately be in position to lift the barbell from the floor. The first repetition on each set, as well as any repetition immediately following an autoregulated break within each set of six repetitions, was required to begin from the floor. To remain consistent with official competition standards [31], any thruster repetition that began from the floor could be performed as a “cluster” (i.e. a clean into a thruster). All subsequent repetitions were initiated from the front rack position. Throughout each workout, a member of the research team was positioned next to the power rack and operated the PERCH software by setting it to record as participants approached the barbell after completing each rowing set. The researcher ended data collection after participants completed six repetitions and transitioned to box jumps. All data was automatically transferred to a cloud database where mean concentric barbell velocity (m х sec^−1^) and power (Watts) on each repetition was organized by date, set, and order of completion. The average, slope, and SD of each barbell velocity and power across all sets on each trial, as well as total repetitions and volume-load completed (total repetitions ×barbell load; in kg), were calculated and retained for comparisons.

A wooden plyo box (0.51 ×0.61 ×0.76-m) was placed at a standardized distance (~5.1 cm) from a pair of in-ground connected force plates (BMS400600 plates, © 2024 Advanced Mechanical Technology, Inc. Watertown, MA). Each device was zeroed according to manufacturer recommendations prior to each workout trial. A researcher operated the force plates at a computer station located on the periphery of the workout area. The force plates were set to record as participants approached the box jump area after completing their sixth barbell thruster repetition, and data collection ended when participants stood at full extension atop the box after their third box jump. All force plate data was collected at a 1000 Hz sampling frequency. Following the completion of each workout trial, the data was transferred via flash drive and stored on a password protected desktop computer to be analyzed by a custom software application developed in LabView (v. Q3, 2023, National Instruments). However, kinetics were only quantified on the second and third jumps on any set. This was because participants were inconsistent in their approach toward the box on the first jump, with some rushing to the force plate to immediately complete the first jump while others would pause. In contrast, the mandatory step down following each repetition ensured that sufficient time was spent standing on the force plate to allow for a clear take-off point to be viewed and quantified on the second and third jumps. The initiation of these jumps was identified on a force-time graph and defined as the moment vertical force began to increase, and values were attained from this point to take-off for analysis. Within this interval, mean and peak force (N) and time to peak force (ms) were quantified and used to calculate rate of force development (RFD; N x sec^−1^) and peak impulse (N x sec) for both jumps, and then these were averaged within each round. The average, slope, and SD of each box jump variable across all sets on each trial, as well as total repetitions and volume-load completed (total repetitions ×average mean force; in kg), were calculated and retained for comparisons.

### 2.6. Dietary intake analyses

Due to the known influence on energy availability and performance, participants were instructed to maintain their normal Caloric intake throughout the course of the investigation. To verify compliance, participants were asked to record all food and beverage intake over the course of the three days leading up to any data collection session, and to follow a similar diet prior to each trial. Food records were collected weekly and entered into a publicly available online database (MyFitnessPal) to determine total kilocaloric (kcals) and macronutrient (g) intake. For statistical analysis, average kcals, carbohydrate, protein, and fat intake over each 3-day period were analyzed relative to body mass across trials.

### 2.7. Statistical analyses

All performance variables (i.e. overall and individual exercise repetition completion rate, volume load completed, and kinetics) were separately assessed across each trial (5S, 5P, 15S, and 15P) using the generalized linear mixed model procedure in SPSS (v.29, Chicago, IL, USA). All models used maximum likelihood estimation and an autoregressive-heterogenous repeated covariance to account for the dependent relationships existing between time points. Sex was added as a factor into the model due to expected pacing and performance differences [3,5,6] despite the common practice of prescribing different rowing Calories, barbell loads, and box jump heights. Any significant F-ratio related to a main effect or interaction involving supplement condition was further investigated by repeating the general linear mixed model procedure using a step-down approach. All data are reported as mean ± standard deviation and statistical significance was set at *p* < 0.05.

## 3. Results

### 3.1. Total workout pacing performance

The time devoted to each workout component by men and women over both supplemental conditions and workout durations are presented in Table 3. A main condition effect was observed for the SD of transition time to rowing (F = 5.8, *p* = 0.020), where transitions were less variable during S (3.23 ± 1.69 seconds) compared to P (3.63 ± 1.58 seconds). A condition ×sex ×duration interaction was observed for the SD of rowing round duration (F = 4.0, *p* = 0.050), though post hoc analysis indicated that this was primarily driven by the main effect of workout duration (*p* < 0.001). Greater variability was seen during the 15-minute (7.36–9.54 seconds) compared to the 5-minute bouts (3.40–4.83 seconds) across supplement conditions in men and women. No other indications of differences between conditions were observed. Otherwise, significant (*p* < 0.05) main effects of duration were noted in the total, average, SD, and slope of time devoted to multiple workout components. No differences were seen between men and women in any of these.

**Table 3.** Time (in seconds) devoted to each workout component.

	Supplement					Placebo				
	5-minute bout			15-minute bout		5-minute bout			15-minute bout	
	Men	Women		Men	Women	Men	Women		Men	Women
Rowing										
Total time	117 ± 16	116 ± 10		345 ± 52	331 ± 40	116 ± 16	118 ± 10		336 ± 39	337 ± 35
Average round	23.48 ± 3.24	23.24 ± 2.03		22.97 ± 3.44	23.82 ± 3.73	23.22 ± 3.22	23.60 ± 1.94		22.4 ± 2.63	22.64 ± 2.40
SD of rounds^†^	3.39 ± 1.70	4.83 ± 3.10		9.15 ± 3.45	7.36 ± 3.35	4.62 ± 2.71	4.18 ± 2.76		9.54 ± 3.76	8.79 ± 3.57
Slope across rounds	−0.28 ± 1.42	−1.12 ± 1.37		−0.09 ± 0.35	0.03 ± 0.52	−0.31 ± 2.08	−0.32 ± 1.26		−0.21 ± 0.26	−0.09 ± 0.32
Rowing break time										
Total time	0 ± 0	0 ± 0		0 ± 0	0 ± 0	0 ± 0	0 ± 0		0 ± 0	0 ± 0
Average round	0.00 ± 0.00	0.00 ± 0.00		0.00 ± 0.00	0.00 ± 0.00	0.00 ± 0.00	0.00 ± 0.00		0.00 ± 0.00	0.00 ± 0.00
SD of rounds	0.00 ± 0.00	0.00 ± 0.00		0.00 ± 0.00	0.00 ± 0.00	0.00 ± 0.00	0.00 ± 0.00		0.00 ± 0.00	0.00 ± 0.00
Slope across rounds	0.00 ± 0.00	0.00 ± 0.00		0.00 ± 0.00	0.00 ± 0.00	0.00 ± 0.00	0.00 ± 0.00		0.00 ± 0.00	0.00 ± 0.00
Transition to Thrusters										
Total time^†^	41 ± 6	42 ± 6		136 ± 29	140 ± 23	41 ± 8	41 ± 5		137 ± 26	140 ± 13
Average transition^†^	8.27 ± 1.27	8.32 ± 1.10		9.09 ± 1.93	9.30 ± 1.51	8.25 ± 1.58	8.26 ± 0.95		9.12 ± 1.73	9.34 ± 0.84
SD of transitions^†^	1.72 ± 0.95	2.75 ± 1.72		5.86 ± 2.88	5.21 ± 2.44	2.21 ± 1.27	2.91 ± 1.86		5.38 ± 2.62	5.84 ± 2.02
Slope across transitions	−0.05 ± 0.60	−0.16 ± 0.70		0.06 ± 0.22	0.03 ± 0.19	0.13 ± 0.59	−0.55 ± 0.93		0.03 ± 0.16	−0.03 ± 0.10
Thrusters										
Total time^†^	58 ± 7	58 ± 7		149 ± 26	163 ± 18	60 ± 7	58 ± 6		148 ± 24	160 ± 18
Average round^†^	11.53 ± 1.49	11.62 ± 1.50		9.94 ± 1.73	10.88 ± 1.22	12.05 ± 1.32	11.54 ± 1.25		9.84 ± 1.59	10.66 ± 1.20
SD of rounds^†^	2.62 ± 1.66	3.75 ± 2.70		5.34 ± 1.87	5.75 ± 2.35	3.03 ± 1.89	4.86 ± 3.50		5.77 ± 1.69	5.58 ± 1.94
Slope across rounds	−0.31 ± 0.71	−0.03 ± 0.82		−0.09 ± 0.14	−0.01 ± 0.13	−0.09 ± 0.91	−0.36 ± 0.74		0.01 ± 0.17	−0.03 ± 0.13
Thrusters break time										
Total time	0 ± 0	1 ± 4		0 ± 0	1 ± 4	0 ± 0	2 ± 6		1 ± 3	12 ± 27
Average round^†^	0.00 ± 0.00	0.24 ± 0.76		0.00 ± 0.00	0.09 ± 0.26	0.00 ± 0.00	0.38 ± 1.20		0.05 ± 0.17	0.81 ± 1.82
SD of rounds	0.00 ± 0.00	0.30 ± 0.95		0.00 ± 0.00	0.34 ± 0.97	0.00 ± 0.00	0.32 ± 1.01		0.19 ± 0.65	1.11 ± 2.21
Slope across rounds	0.00 ± 0.00	0.19 ± 0.6		0.00 ± 0.00	0.02 ± 0.07	0.00 ± 0.00	0.16 ± 0.51		0.00 ± 0.01	0.11 ± 0.21
Transition to Box Jumps										
Total time^†^	19 ± 5	22 ± 4		76 ± 29	69 ± 11	21 ± 6	21 ± 3		84 ± 31	70 ± 18
Average transition^†^	3.75 ± 0.91	4.32 ± 0.89		5.08 ± 1.92	4.60 ± 0.70	4.17 ± 1.21	4.10 ± 0.50		5.62 ± 2.04	4.70 ± 1.21
SD of transitions^†^	1.42 ± 0.68	1.99 ± 1.64		3.69 ± 2.52	3.04 ± 1.03	1.66 ± 1.00	1.86 ± 0.91		3.84 ± 2.72	3.25 ± 1.45
Slope across transitions	−0.06 ± 0.42	0.13 ± 0.42		0.05 ± 0.13	0.05 ± 0.07	−0.08 ± 0.50	0.01 ± 0.42		0.06 ± 0.18	0.00 ± 0.06
Box Jumps										
Total time^†^	30 ± 5	28 ± 3		83 ± 14	77 ± 8	30 ± 4	27 ± 3		82 ± 10	74 ± 7
Average round^†^	6.07 ± 1.04	5.54 ± 0.65		5.56 ± 0.94	5.11 ± 0.56	5.98 ± 0.86	5.40 ± 0.57		5.46 ± 0.69	4.94 ± 0.49
SD of rounds^†^	2.30 ± 1.05	2.36 ± 1.42		3.60 ± 1.50	3.03 ± 0.81	2.62 ± 1.58	2.39 ± 1.15		3.47 ± 1.13	3.27 ± 0.95
Slope across rounds^†^	0.28 ± 0.55	0.48 ± 0.57		0.02 ± 0.08	0.05 ± 0.06	0.21 ± 0.92	0.31 ± 0.71		0.05 ± 0.06	0.05 ± 0.06
Box Jumps break time										
Total time	0 ± 0	0 ± 0		0 ± 0	0 ± 0	0 ± 0	0 ± 0		0 ± 0	0 ± 0
Average round	0.00 ± 0.00	0.00 ± 0.00		0.00 ± 0.00	0.00 ± 0.00	0.00 ± 0.00	0.00 ± 0.00		0.00 ± 0.00	0.00 ± 0.00
SD of rounds	0.00 ± 0.00	0.00 ± 0.00		0.00 ± 0.00	0.00 ± 0.00	0.00 ± 0.00	0.00 ± 0.00		0.00 ± 0.00	0.00 ± 0.00
Slope across rounds	0.00 ± 0.00	0.00 ± 0.00		0.00 ± 0.00	0.00 ± 0.00	0.00 ± 0.00	0.00 ± 0.00		0.00 ± 0.00	0.00 ± 0.00
Transition to Rowing										
Total time^†^	35 ± 4	34 ± 6		110 ± 12	109 ± 27	32 ± 5	34 ± 7		113 ± 11	106 ± 24
Average transition^†^	6.90 ± 0.76	6.72 ± 1.27		7.36 ± 0.83	7.24 ± 1.79	6.33 ± 1.02	6.70 ± 1.39		7.51 ± 0.75	7.07 ± 1.59
SD of transitions^†§^	1.84 ± 0.65	2.61 ± 1.59		4.56 ± 1.35	4.08 ± 1.51	2.52 ± 1.26	3.06 ± 1.52		4.69 ± 1.21	4.32 ± 1.33
Slope across transitions^†^	0.42 ± 0.78	0.51 ± 0.66		0.05 ± 0.15	0.04 ± 0.08	0.13 ± 0.80	0.73 ± 0.77		0.06 ± 0.06	0.07 ± 0.06

† = Significant (*p* < 0.05) difference in workout duration; ‡ = Significant (*p* < 0.05) difference between men and women; § = Significant (*p* < 0.05) difference favoring supplement condition.

**Figure 2.**
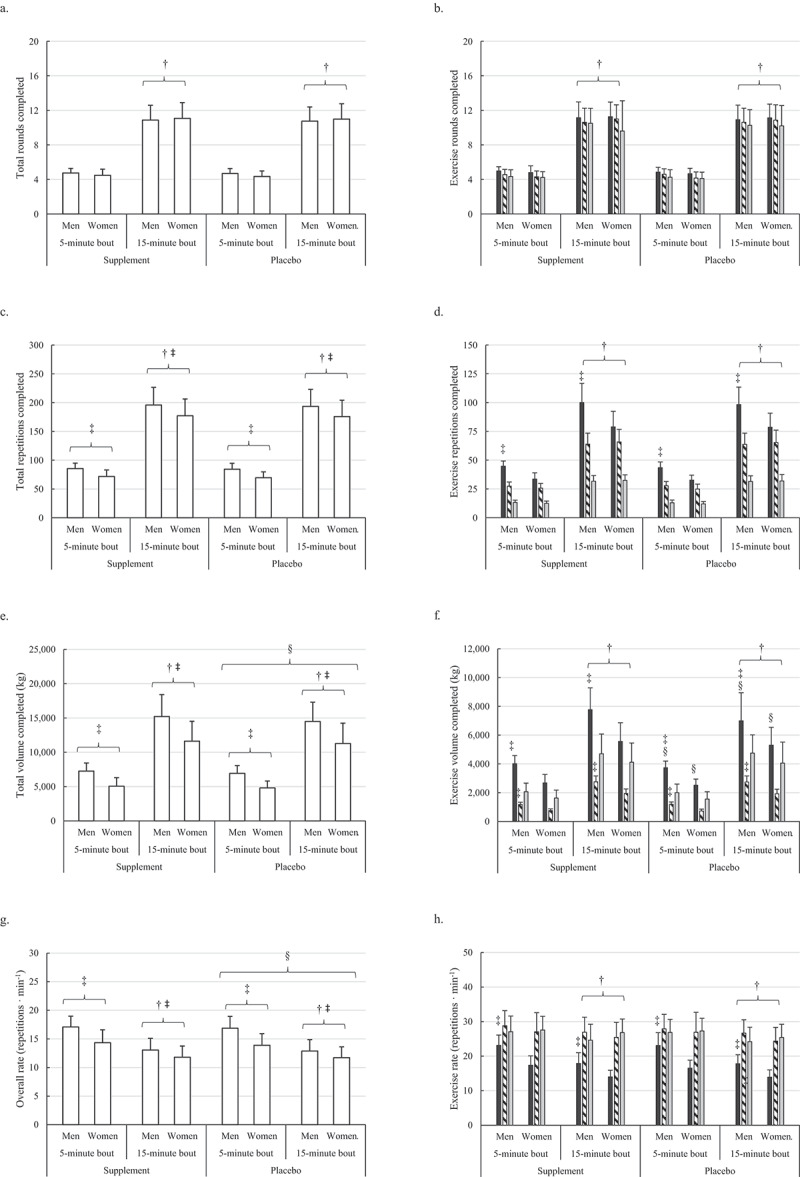
Sex, workout duration, and supplemental condition comparisons for the rounds completed overall and per exercise (a & b), repetitions completed overall and per exercise (c & d), volume completed overall and per exercise (e & f), and overall and per exercise repetition completion rate (g & h).

Main condition effects were also noted for total volume load (F = 7.6, *p* = 0.009), rowing volume load (F = 7.4, *p* = 0.009), and total repetition completion rate (F = 4.3, *p* = 0.043). Regardless of workout duration and sex, participants completed a greater total volume load during S (9,922 ± 4,583 kg) compared to P (9,501 ± 4,364 kg), and specifically, a greater rowing volume load during S (5,084 ± 2,195 kg) compared to P (4,703 ± 2,077 kg). Overall workout repetition completion rate was also faster during S (14.2 ± 2.8 repetitions ×min^−1^) compared to P (13.9 ± 2.7 repetitions ×min^−1^), which may partially be explained by the trend (F = 3.3, *p* = 0.077) noted for the faster thruster repetition completion rate during S (27.2 ± 4.6 repetitions ×min^−1^) compared to P (26.6 ± 4.5 repetitions ×min^−1^). No other differences involving supplement condition were noted for measures of total workout performance. On a side note, significant main effects for sex (*p* < 0.05) and workout duration were also noted for multiple pacing variables. Differences between men and women across workout durations and supplement conditions in total workout quantifiers are illustrated in Figure 2

A.† = Significant (*p* < 0.05) difference in workout duration; ‡ = Significant (*p* < 0.05) difference between men and women; § = Significant (*p* < 0.05) difference favoring supplement condition. Overall performance (white bars), rowing performance (black bars), thrusters performance (striped bars), and box jump performance (gray bars).

### 3.2. Rowing performance

A main effect for condition was only seen with average watts generated across rowing rounds (F = 8.4, *p* = 0.005). Regardless of sex and workout duration, participants rowed with greater power during S (243 ± 82 W) compared to P (227 ± 78 W). No other differences related to supplement condition were found. Main effects of sex were noted for rowing Calories per stroke (average: F = 19.4, *p* < 0.001; SD: F = 4.6, *p* = 0.043) and average rowing power (F = 22.0, *p* < 0.001). Men averaged approximately 0.2 more Calories per stroke and produced ~ 44.2% more power across each round, though women were more consistent in calories per stroke. Meanwhile, main effects of duration were found with rowing Calories per stroke (average: F = 14.5, *p* < 0.001; slope: F = 26.3, *p* < 0.001), rowing power (average: F = 90.5, *p* < 0.001; slope: F = 6.8, *p* < 0.001), and average rowing strokes per minute (F = 25.3, *p* < 0.001). Participants rowed ~ 8.3% more Calories per stroke at a faster pace (~4.3 strokes per minute) and produced approximately ~ 47.2% more power across rounds of the 5-minute bouts compared to the 15-minute bouts, but experienced steeper declines in Calories per stroke (~226%) and power (~175%). Rowing kinetics are presented in Table 4.

**Table 4.** Sex, workout duration, and supplemental condition comparisons for rowing and thrusters performance.

	Supplement							Placebo						
	5-minute bout				15-minute bout			5-minute bout				15-minute bout		
	Men		Women		Men		Women	Men		Women		Men		Women
Rowing calories (per stroke)														
Average^†‡^	0.73 ± 0.09		0.57 ± 0.10		0.66 ± 0.11		0.50 ± 0.08	0.71 ± 0.11		0.56 ± 0.08		0.69 ± 0.09		0.50 ± 0.08
Standard Deviation^‡^	0.06 ± 0.03		0.06 ± 0.02		0.09 ± 0.04		0.05 ± 0.02	0.07 ± 0.03		0.06 ± 0.03		0.07 ± 0.05		0.04 ± 0.02
Slope^†^	−0.03 ± 0.02		−0.03 ± 0.02		−0.01 ± 0.01		−0.01 ± 0.01	−0.03 ± 0.03		−0.03 ± 0.02		−0.01 ± 0.01		0.00 ± 0.01
Rowing power (W)														
Average^†‡§^	341 ± 65		226 ± 48		224 ± 49		165 ± 35	321 ± 60		210 ± 34		205 ± 56		156 ± 39
Standard Deviation	54.3 ± 37.4		35.2 ± 15.3		44.7 ± 30.5		32.8 ± 11.3	62.4 ± 48.8		32.1 ± 15.0		52.2 ± 54.4		30.9 ± 13.0
Slope^†^	−26.9 ± 28.1		−17.7 ± 14.2		−10.5 ± 10.6		−6.6 ± 4.2	−32.0 ± 34.9		−16.8 ± 12.8		−11.0 ± 13.9		−5.9 ± 5.0
Rowing strokes (per minute)														
Average^†^	32.6 ± 5.1		30.2 ± 4.4		28.3 ± 4.4		27.9 ± 3.3	32.4 ± 5.1		30.1 ± 2.8		25.1 ± 4.4		27.3 ± 3.6
Standard Deviation	2.44 ± 0.98		1.72 ± 0.90		2.37 ± 1.29		2.13 ± 0.81	2.62 ± 2.00		1.60 ± 0.84		2.66 ± 1.99		1.95 ± 0.80
Slope	−0.58 ± 1.23		−0.36 ± 0.69		−0.16 ± 0.57		−0.19 ± 0.43	−0.78 ± 1.77		−0.17 ± 0.89		−0.24 ± 0.55		−0.25 ± 0.31
Thrusters velocity (m · sec^−1^)														
Average^‡^	1.27 ± 0.17		1.10 ± 0.17		1.24 ± 0.16		1.10 ± 0.15	1.25 ± 0.17		1.09 ± 0.17		1.23 ± 0.17		1.12 ± 0.16
Standard Deviation^†^	0.07 ± 0.04		0.06 ± 0.02		0.04 ± 0.01		0.05 ± 0.03	0.06 ± 0.03		0.06 ± 0.03		0.04 ± 0.02		0.05 ± 0.02
Slope^†^	−0.04 ± 0.02		−0.02 ± 0.03		0.00 ± 0.01		0.00 ± 0.01	−0.03 ± 0.03		−0.02 ± 0.02		0.00 ± 0.01		0.00 ± 0.01
Thrusters power (W)														
Average^‡^	537 ± 71		318 ± 49		523 ± 67		317 ± 42	529 ± 71		319 ± 53		520 ± 71		323 ± 47
Standard Deviation^†^	29.5 ± 15.3		16.4 ± 7.1		17.1 ± 5.7		13.7 ± 8.2	26.4 ± 11.6		24.7 ± 29.0		17.8 ± 8.2		13.8 ± 6.7
Slope^†^	−16.0 ± 9.5		−5.4 ± 7.9		−1.5 ± 3.5		−1.4 ± 2.0	−11.0 ± 14.0		−9.5 ± 20.8		−1.4 ± 4.4		−0.4 ± 2.0

† = Significant (*p* < 0.05) difference in workout duration; ‡ = Significant (*p* < 0.05) difference between men and women; § = Significant (*p* < 0.05) difference favoring supplement condition.

### 3.3. Thruster performance

While a trend for a faster barbell thruster repetition completion rate was noted during S (~2.3%, *p* = 0.077), no significant differences related to supplement condition were seen with barbell thruster kinetics. Main effects of sex were seen for average barbell thruster velocity (F = 5.0, *p* = 0.036) and power (F = 74.1, *p* < 0.001), where men performed repetitions with greater velocity (men = 1.25 ± 0.16 m·sec^−1^, women = 1.10 ± 0.16 m·sec^−1^) and power (men = 527 ± 68 W, women = 319 ± 46 W) despite being assigned a greater absolute load and regardless of supplement condition or workout duration. Main effects of workout duration were also noted for the SD and slope of barbell velocity (F = 9.1–22.3, *p* < 0.05) and power (F = 8.6–16.1, *p* < 0.05) across rounds, and showed greater variability and a steeper decline during the 5-minute workouts. Barbell thruster kinetics are presented in Table 4.

### 3.4. Box jump performance

A sex × condition interaction was noted with the SD in box jump mean force across rounds (F = 4.3, *p* = 0.042), where men were ~ 69.7% more variable in their force expression during S (*p* = 0.013) and ~ 30.5% more variable during S compared to P (*p* = 0.009). No differences between supplemental conditions were seen in women. Main effects of condition were also found with the SDs of box jump peak force (F = 15.2, *p* < 0.001), time to peak force (F = 6.6, *p* = 0.030), and RFD (F = 5.1, *p* = 0.027). In each case, greater variability was noted in S compared to P (~19.0–30.2%, *p* < 0.05). Box jump kinetics are presented in Table 5.

**Table 5.** Sex, workout duration, and supplemental condition comparisons for box jump performance.

	Supplement					Placebo				
	5-minute bout			15-minute bout		5-minute bout			15-minute bout	
	Men	Women		Men	Women	Men	Women		Men	Women
Mean force (N)										
Average^†‡^	1504 ± 221	1278 ± 250		1438 ± 256	1225 ± 256	1494 ± 213	1243 ± 245		1464 ± 214	1216 ± 255
Standard Deviation^‡^	83.7 ± 41.1^‡§^	50.4 ± 25.6		75.6 ± 43.7^‡§^	43.1 ± 18.7	61.1 ± 43.1	45.2 ± 26.2		60.3 ± 29.3	46.1 ± 29.1
Slope^†^	−39.0 ± 32.9	−12.1 ± 36.4		−14.3 ± 70.8	1.9 ± 32.1	−25.6 ± 34.8	−18.9 ± 13.2		−3.2 ± 12.0	10.7 ± 34.1
Peak force (N)										
Average^‡^	2732 ± 359	2307 ± 522		2688 ± 402	2214 ± 503	2713 ± 355	2247 ± 493		2720 ± 339	2214 ± 515
Standard Deviation^#^	174 ± 73	146 ± 72		131 ± 40	123 ± 61	119 ± 53	105 ± 44		103 ± 40	117 ± 57
Slope^†‡^	−87.5 ± 94.1	−25.1 ± 90.9		−22.4 ± 76.6	15.9 ± 96.3	−33.2 ± 66.1	−31.3 ± 37.4		−11.4 ± 41.0	29.7 ± 84.2
Time to peak force (ms)										
Average^‡^	258 ± 75	181 ± 51		253 ± 92	187 ± 47	259 ± 72	186 ± 52		272 ± 64	177 ± 55
Standard Deviation‡^#^	31.7 ± 11.6	17.2 ± 13.7		33.6 ± 13.1	20.7 ± 10.4	25.1 ± 11.8	17.0 ± 9.2		29.9 ± 13.2	15.5 ± 7.6
Slope	10.6 ± 17.7	−2.2 ± 11.6		2.0 ± 23.9	−2.2 ± 10.0	3.5 ± 15.5	4.4 ± 7.5		−1.7 ± 4.5	−2.0 ± 6.6
RFD (N · sec^−1^)										
Average	10261 ± 3807	12773 ± 5369		10999 ± 4962	11570 ± 4650	10144 ± 4251	11870 ± 4655		9360 ± 3057	12742 ± 5703
Standard Deviation^#^	1976 ± 1215	1838 ± 1087		2109 ± 1394	1913 ± 913	1519 ± 1200	1504 ± 876		1482 ± 865	2000 ± 1509
Slope^†^	−788 ± 1218	−188 ± 1219		−239 ± 1307	297 ± 1302	−232 ± 1210	−349 ± 501		34 ± 479	318 ± 1034
Impulse (N · sec)										
Average^‡^	372 ± 109	219 ± 50		354 ± 143	219 ± 62	373 ± 108	222 ± 66		382 ± 97	209 ± 79
Standard Deviation‡	29.2 ± 12.1	17.1 ± 15.4		31.8 ± 17.0	18.7 ± 10.3	26.6 ± 14.4	16.4 ± 15.8		29.6 ± 13.2	15.8 ± 10.2
Slope	6.41 ± 19.11	−3.45 ± 11.83		2.83 ± 10.09	−3.16 ± 7.04	−1.85 ± 17.82	0.54 ± 5.34		−2.80 ± 7.63	−1.24 ± 8.19

† = Significant (*p* < 0.05) difference in workout duration; ‡ = Significant (*p* < 0.05) difference between men and women; § = Significant (*p* < 0.05) difference favoring supplement conditions; # = Significant (*p* < 0.05) difference favoring placebo conditions.

### 3.5. Caloric and macronutrient intake

A sex ×condition interaction was found for absolute protein intake (F = 4.3, *p* = 0.046), where men generally consumed more protein than women prior to S (*p* < 0.05) and men consumed more protein prior to S compared to P (*p* = 0.041). Additionally, main effects of sex were noted for total Caloric (F = 19.0, *p* < 0.001) and absolute carbohydrate (F = 7.4, *p* = 0.012) intake. However, these differences were no longer present between supplement conditions when caloric and macronutrient intake were made relative to body mass. Table 6 presents absolute and relative Caloric and macronutrient intake across all experimental trials.

**Table 6.** Sex, workout duration, and supplemental condition comparisons for absolute and relative caloric and macronutrient intake.

	Supplement					Placebo				
	5-minute bout			15-minute bout		5-minute bout			15-minute bout	
	Men	Women		Men	Women	Men	Women		Men	Women
Calories (kCal)										
Absolute^‡^	3244 ± 1782	1800 ± 350		2846 ± 595	1844 ± 380	2674 ± 448	1807 ± 457		2689 ± 681	1832 ± 502
Relative (per kg)	37.1 ± 20.3	26.3 ± 6.1		32.8 ± 9.5	26.4 ± 3.8	30.8 ± 7.9	26.2 ± 7.1		31 ± 10.2	26.6 ± 8.2
Carbohydrate (g)										
Absolute^‡^	261 ± 115	189 ± 65		305 ± 111	255 ± 223	279 ± 62	178 ± 74		277 ± 104	175 ± 75
Relative (per kg)	3.04 ± 1.51	2.78 ± 1.09		3.52 ± 1.46	3.38 ± 2.14	3.22 ± 0.96	2.57 ± 1.08		3.23 ± 1.46	2.57 ± 1.23
Fat (g)										
Absolute	105.8 ± 30.1	65.6 ± 17.1		108.8 ± 29.3	71.7 ± 10.8	107.5 ± 25.1	78.5 ± 32.3		101.4 ± 39	72.7 ± 21.2
Relative (per kg)	1.21 ± 0.42	0.96 ± 0.3		1.26 ± 0.44	1.04 ± 0.22	1.24 ± 0.4	1.17 ± 0.6		1.17 ± 0.53	1.06 ± 0.36
Protein (g)										
Absolute^‡^	153.5 ± 38.6^§^	116.6 ± 35.1		151.3 ± 18.4§	111.3 ± 32.8	127.1 ± 24.4	148.4 ± 93.9		143.8 ± 21	112.1 ± 34.5
Relative (per kg)	1.74 ± 0.48	1.66 ± 0.38		1.73 ± 0.32	1.58 ± 0.34	1.44 ± 0.24	2.26 ± 1.89		1.63 ± 0.25	1.59 ± 0.36

† = Significant (*p* < 0.05) difference in workout duration; ‡ = Significant (*p* < 0.05) difference between men and women; § = Significant (*p* < 0.05) difference favoring supplement conditions.

## 4. Discussion

The purpose of this study was to examine the acute effects of MIPS and exercise duration on HIFT workout performance. Experienced men and women volunteered to complete 5- and 15-minute versions of the same AMRAP-style workout after consuming MIPS or placebo and were evaluated based on their pacing strategy and overall repetitions and volume load completed, as well as on volume load completed and kinetics expressed on each exercise across AMRAP rounds. The present MIPS formulation was hypothesized to allow for greater volume-load to be completed and a more consistent expression of kinetics. The hypothesis was based on a previous study that demonstrated improved resistance exercise performance from this formulation [20], while considering the suitability of its ingredients toward facilitating optimal HIFT pacing strategies [4–6,13]. The data partially supported this hypothesis. Regardless of AMRAP duration, both men and women completed greater total volume load at a faster pace after consuming the supplement. More specifically, more consistent transitions to rowing, greater rowing power, and a tendency for faster barbell thruster repetitions were seen during S compared to P. However, box jump force expression was less consistent during the supplement conditions. Although another study examined HIFT performance adaptations in response to chronic ingestion of another MIPS formulation [22], this is the first study to examine the acute benefits of any formulation on HIFT workout performance.

Workout performance was described by repetition completion rate and volume load completed because daily variation in WOD composition makes the typical score (i.e. repetitions completed, time-to-completion) in one workout irrelevant to others [4]. These metrics account for differences in how each type of exercise modality is quantified and programmed for men and women [1–3,31,33] and organizes them into the same relative units to enable fair comparisons [4]. The importance of evaluating HIFT performance this way was made apparent when differences in repetition completion rate and volume load completed were seen between supplement conditions but not with repetitions completed. The differences in prescription assigned to men and women would have led to more variability in how often men and women transitioned between exercises and in the amount of work represented by each completed repetition. Those differences would have remained unaccounted for in any subsequent statistical comparison if performance were solely based on the number of repetitions completed. By including repetition completion rate and volume load as additional metrics of performance, the influence of prescription differences in rowing calories, barbell thruster load, and box jump height is controlled. Subsequent comparisons revealed a faster repetition completion rate and greater volume load completed during S, and this was primarily the result of more powerful rowing strokes. Future HIFT studies will be able to make more direct comparisons to these findings by using a similar method to quantify WOD performance.

The improved performances seen with MIPS were driven by enhanced rowing performance, not barbell thrusters or box jumps, and this may be due to the number of repetitions assigned to each exercise. Maintaining a consistent effort is thought to be a key strategy for maximizing HIFT workout performance [4–6,13]. From a physiological standpoint, the magnitude of a sustained effort depends on the availability of energy to working muscles and the accumulation of fatigue throughout the expected duration. The specific MIPS formulation from this study was selected because it contained several ingredients known to facilitate energy substrate mobilization and delivery [18,35–39] and their ability to limit the disruptive effects of metabolic byproducts (i.e. hydrogen) [35,37]. However, these effects had not yet been observed in HIFT. Unlike more traditional exercise forms (i.e. resistance training, continuous aerobic exercise, high-intensity interval training), HIFT does not usually prescribe specific rest periods aside from mandated transitions between exercises [1–3,31,33]. Otherwise, trainees are free to break whenever and as often as needed. This study’s WOD mandated transitions after nine or seven rowing Calories, six barbell thrusters, and three box jumps, and breaks were only taken by participants during barbell thrusters. This resulted in the most time (overall and continuously) being devoted to rowing (~28.6 seconds per round). Previous studies noted enhanced performance in continuous maximal efforts lasting 30 seconds after participants consumed various ingredients found in the present MIPS formulation [38–40], as well as another MIPS formulation [26]. Although “all out” effort like that in a Wingate test was unlikely to have been given in this WOD, except possibly when time was about to expire, the expected and unbroken duration of repeated rowing sets may have placed a similar glycolytic energy demand [16]. The data suggests that S may have done better than P in supplying requisite energy to sustain more powerful rowing strokes.

Conversely, the average durations of six barbell thrusters using a load equating to approximately ~ 50% of 1-RM (~13.8 seconds per round) and three box jumps (~7.4 seconds per round) were within the expected capacity of the phosphagen energy system [16]. Moreover, the nature of both exercises provided several official and unofficial breaking opportunities. All official breaks were observed during barbell thrusters, whereas unofficial breaks could be seen at the completion of both thrusters (e.g. pausing with barbell locked out above the head) and box jump repetitions (e.g. pausing when standing atop the box, taking one’s time stepping down from the box). Unofficial breaks would have helped maintain repetition completion rate while limiting the accumulation of fatigue from truly continuous movement or from taking official breaks (or irregular changes in effort) [16]. In either case, breaks were uncommon within the present WOD design, and this may have limited the potential benefits of the present MIPS formulation in this study. An interesting follow-up would be to determine whether acute supplementation is more useful when workouts are comprised of larger repetition-sets that either require participants to sustain continuous effort over a longer period or break more frequently.

Though an exception was seen where box jump force expression was more variable during S, the data did not support the hypothesis that MIPS supplementation would lead to a more consistent expression of kinetics. It is not clear whether this outcome has a physiological or strategic explanation or if it was a spurious consequence of a methodological limitation. The WOD’s box height averaged out to be approximately 113% of the participants’ maximal vertical jump height and would thus have required a leg tuck to land in a squat position before standing erect to complete each repetition. Intentionally jumping higher would increase force expression and energy requirements [41] but also reduce effort to stand erect atop the box. Fatigue could have led to the greater variability seen during S, but it is odd that it occurred during the briefest movement and nowhere else. If this hypothesis is correct, the greater variation seen during S may have been a reactive or proactive response to current or future energy demands. Much like how greater heart rate variability is thought to reflect an autonomic response to inconsistent stressors of an environment [42], participants may have purposefully modulated their jumping strategy to account or prepare for greater effort given elsewhere. Varying effort on each 3-repetition set of a low-intensity plyometric would have been less impactful to overall energy demands and performance than pausing with (or completely dropping) a loaded barbell during thrusters. This could have allowed participants to be more consistent in executing their transition strategy to rowing and putting forth greater effort in the most time-consuming workout component. Alternatively, it is possible that this observation is an example of committing type 1 error. With kinetics only being considered on two of three jumps per set, the calculated averages and SDs across rounds were inherently more volatile than the kinetics derived from complete repetition-sets of thrusters and rowing. The impact of each individual jump would have a more drastic effect on the aggregated scores used for analysis. These effects can be minimized or negated when more repetitions are assigned per set. Future studies should consider both possibilities when attempting to support or refute these findings.

Women were hypothesized to respond better to the supplement because they weighed ~18.9 kg less than men and received a higher relative dose of the standard MIPS formulation. However, no sex differences were noted with respect to the supplement condition, and this may have been because several ingredients were sufficiently dosed to have an acute effect for men in this study [18,35–39]. Instead, the only sex differences seen in this study were expected and partially agree with previous comparisons between equally ranked men and women [3,5,6,43]. Despite modified programming, men completed repetitions at a faster rate and averaged a greater expression of force, velocity, or power on each exercise, and their performance was best exemplified by their superior rowing repetition completion rate. Rowing has been the most common monostructural exercise to appear in HIFT competition [43], and it is an important skill to develop aptitude in for HIFT athletes. Efficient technique may be learned within a few months of devoted attention [44], but from there, physical attributes will dictate performance [45]. The participants in this study averaged approximately 6 years of HIFT experience and would have had plenty of opportunities to develop rowing skills, leaving the physical advantages of men (e.g. height, strength) as the most likely explanations for their faster rowing pace. Nevertheless, their greater expression of barbell thruster (velocity and power) and box jump (force and impulse) kinetics did not directly translate to faster repetition completion rates. In these, it is possible that their advantages were offset by women generally being more consistent in their pacing and expression of kinetics. Though men had been previously shown to be more consistent [5], the ability to maintain consistent pacing over an entire WOD is a learned skill, and despite having a statistically similar number of years of HIFT experience, women reported having ~ 1.4 times more years of regular (at least 2 sessions per week for more than 8 months in a year) training experience. The nature of HIFT experience has a greater impact on performance than years of experience alone [46], and frequent training for a longer period might explain why this group of women displayed more consistent pacing. Their consistency, or the greater variability in men, may also have masked potential differences related to supplement condition.

The differences observed between workout durations were also consistent with a previous report about pacing strategies across five different competition WODs [5]. Participants spent more time on each exercise, completed repetitions at a slower rate, expressed diminished kinetics, and were often more variable during the 15-minute bouts, though steeper declines (i.e. slope) were noted in the 5-minute bouts. Shorter duration HIFT workouts ( < 5 minutes) that consist of high-intensity movements performed at an unsustainably fast pace are often characterized as “sprints.” For example, athletes must complete ~ 16–22 repetitions per minute to complete 45 barbell thrusters and pull-ups within 4–5 minutes to earn a 50^th^ percentile score in the benchmark WOD “Fran” [33]. Meanwhile, 50^th^ percentile scores in the 15–20-minute AMRAP’s of the 2021–2022 CrossFit® Opens averaged approximately 12 repetitions per minute [3]. The 5- (~15.5 repetitions per minute) and 15-minute (12.4 repetitions per minute) bouts in this study mirrored those repetition completion rates. However, these observations were not affected by supplemental condition. It had been hypothesized that the fast-paced 5-minute bouts would not be long enough to allow a meaningful benefit from enhanced blood-flow and nutrient delivery to exercising muscle [16]. The short bouts would more heavily rely on anaerobic metabolic pathways, and consequently, fatigue management (e.g. muscle buffering capacity) would be the most advantageous physiological trait. Although the MIPS formulation contained ingredients known to affect muscle buffering capacity (e.g. beta alanine, creatine monohydrate), their effects are typically realized after chronic supplementation [35,37]. Still, effects have been reported after a single dose [35,37] and the impact of caffeine on repeated high-intensity efforts cannot be discounted [36]. Regardless, the addition of 10 minutes to the present HIFT circuit did not create a large enough difference in pace that would reveal differential effects from the supplement.

There were notable limitations present in this study that may have affected the context of these results. Vague and inadequate descriptions about sample populations are common in HIFT research because there are numerous skills required to be considered elite in the training strategy. Some studies have used all-encompassing metrics like official competition ranks [3,5,6,47–49], but most settle on 6–12 months of experience (i.e. “trained”) or no experience [1]. This study excluded elite athletes (i.e. no participant had ever advanced beyond the CrossFit® Open when they enrolled in this study) due to their limited availability but extended the experience requirement to two years and asked participants about their training history. Unfortunately, there were few common experiences beyond those reported in Table 1 that could be used to evaluate skill differences amongst participants. Their unique experiences may have contributed to increased variability (i.e. “noise”) seen among dependent variables and masked by additional differences. Additional variability might be attributed to the adequacy of the placebo that was selected for this study. Although the placebo was similar to the supplement in appearance, flavor, and Calorie content, participants were able to correctly identify whether they had consumed the supplement or placebo approximately 78% of the time both before and after each workout. Their correct guesses were most commonly based on the difference in “texture” and a “tingling feeling” (presumably paresthesia from beta alanine). With both beta alanine and caffeine being known to cause familiar sensations [35,36], future studies involving MIPS containing these ingredients should consider placebo alternatives that match these sensations. Nevertheless, the amount of bias this introduced may have been mitigated by doubt. Participants switched their answers post-exercise approximately 40% of the time, and their guesses were evenly split between being correct prior to exercise and then guessing wrong and vice versa. Since they remained ignorant about the accuracy of their choices until after the study, it is possible that sufficient doubt was present to limit how much sway bias had on the results. Finally, the generalizability of the MIPS effect on the specific WODs selected for this study may also be limited. Regardless of the reasons for selection, the two WODs were drawn from infinite possible combinations [1,2], and a different combination might not elicit the same results. To aid in the generalizability of our findings and comparisons with follow-up investigations, the present data were analyzed and reported using previously suggested techniques (i.e. repetition completion rate, volume load) for equating HIFT-style workouts [4].

This study is the first to assess the effects of acute supplementation of any MIPS formulation on pacing and the kinetics expressed throughout a HIFT-style workout. The data showed that supplementation led to a faster repetition completion rate and the completion of a greater volume load, primarily through more powerful rowing. The data further suggested that these enhancements were not affected by workout duration and occurred similarly in men and women. Although MIPS did not affect the typical AMRAP score (i.e. repetitions completed), the observed enhancements may be indicative of a benefit for specific types of prescription within HIFT-style WODs. Each round of rowing was two and four times as long as each round of thrusters and box jumps, respectively. Unlike box jumps and thrusters, where participants could take breaks and still finish sets more quickly, no breaks were taken during rowing. This meant that average stroke rate and power determined round duration. Given the mechanisms of action for its various ingredients, it is possible that the MIPS formulation from this study is more beneficial for workouts and workout components that demand sustained efforts for longer durations and less frequent breaks and/or transitions. Future investigations should seek to confirm this hypothesis, and to also test the present formulation against various sub-combinations of its individual ingredients. Regardless of whether subsequent studies use the same, similar, or completely different WODs, they will be aided by our method of reporting. Performances were decomposed into repetition completion rate and volume load completed to enable workload comparisons. Thus, our findings may be generalized to specific pacing-workload combinations and not be limited to a specific WOD.

## Data Availability

The data that support the findings of this study are available on request from the corresponding author, [GTM]. The data are not publicly available due to their containing information that could compromise the privacy of research participants.
